# Alternative Sigma Factors of RNA Polymerase as Master Regulators in the Pathogenic Spirochaete *Leptospira interrogans*

**DOI:** 10.3390/pathogens14111100

**Published:** 2025-10-29

**Authors:** Sabina Kędzierska-Mieszkowska, Zbigniew Arent

**Affiliations:** 1 Department of General and Medical Biochemistry, Faculty of Biology, University of Gdansk, 80-308 Gdansk, Poland; 2 Department of Infectious Diseases and Public Health, Faculty of Veterinary Medicine, University of Agriculture in Krakow, 30-248 Krakow, Poland; zbigniew.arent@urk.edu.pl

**Keywords:** adaptive response, gene regulation, *Leptospira interrogans*, sigma factors, transcription, virulence

## Abstract

This review summarizes the current knowledge on the role of alternative σ factors in the highly invasive spirochaete *Leptospira interrogans*, the causative agent of leptospirosis. This globally distributed zoonosis affects both animals and humans, resulting in substantial public health and economic consequences. Together with the primary σ^70^, alternative σ factors provide transcriptional flexibility essential for bacterial adaptation to environmental changes and host–pathogen interactions. Comparative genomic analyses have revealed that the *L. interrogans* genome encodes 14 σ factors, including one housekeeping σ^70^-like factor and three types of alternative σ factors: σ^54^, σ^28^, and 11 predicted extracytoplasmic function (ECF, σᴱ-type) factors. This review discusses the characteristics of these regulators, with particular emphasis on the poorly understood ECF σ factors and their potential roles in gene regulation, adaptive responses, and pathogenicity.

## 1. Introduction

The *Leptospira* genus (*Spirochaetes* phylum) is comprised of pathogenic, intermediate, and saprophytic species. Saprophytic species are free-living and primarily inhabit soil or surface water [1], whereas pathogenic species can infect a wide range of animals and humans. Intermediate species display moderate pathogenicity in both animals and humans [2]. One of the pathogenic species is the highly invasive *Leptospira interrogans*, which causes leptospirosis, a zoonotic disease that has significant public health and economic impacts worldwide [3,4,5]. This disease is responsible for an estimated one million human cases and about 60,000 deaths annually [6,7]. In livestock, the economic losses are largely driven by reproductive disorders, abortion, reduced fertility, and decreased milk production, while in companion and wild animals, the disease can cause a severe systemic illness [8,9,10,11]. In humans, transmission mainly occurs through direct contact with the urine of infected animals or indirectly via contaminated water or moist soil, reflecting a strong environmental link to this disease [12]. Following host entry, *Leptospira* disseminates rapidly through the bloodstream and can colonize multiple organs. Clinical presentation ranges from mild flu-like symptoms to life-threatening forms, such as Weil’s syndrome, which is characterized by jaundice, renal and hepatic failure, pulmonary hemorrhage, and in some cases death [4,13]. Despite the global burden of leptospirosis, the molecular mechanisms underlying *L. interrogans* virulence and disease pathogenesis remain incompletely understood, primarily due to limitations in the genetic manipulation of pathogenic strains. However, recent advances in genetic and genomic tools have begun to provide new insights into these processes [14,15].

The complex life cycle of *L. interrogans* includes environmental persistence, shedding from chronically infected animals, host acquisition, and dissemination to the kidneys [4]. To survive, these bacteria must cope with diverse environmental and host-induced stressors, including iron limitation, temperature and pH changes, osmotic stress, and host immune defenses. These adaptive responses are largely mediated by transcriptional regulators, among which alternative σ factors play a central role by directing RNA polymerase to specific promoters and orchestrating global transcriptional programs [16,17].

In *L. interrogans*, comparative genomics and in silico genome-wide analyses have revealed a repertoire of 14 σ factors (Table 1), including one σ^70^-like housekeeping factor responsible for maintaining basal transcription necessary for growth, as well as a set of alternative σ factors: σ^54^, σ^28^, and 11 ECF σᴱ-type, which collectively regulate adaptive responses and contribute to this bacterium’s pathogenic potential [14,15]. Interestingly, unlike many other bacteria, *L. interrogans* lacks the RpoH (σ^H^/σ^32^) factor, which is essential for heat shock responses, as well as the RpoS (σ^S^/σ^38^) factor, a key regulator of general stress responses.

This review summarizes current knowledge on alternative σ factors in *L. interrogans*, with particular emphasis on the ECF σ factors (ECF σs), which represent the largest and most poorly understood group of transcriptional regulators. It also discusses their potential roles in adaptive responses and host–pathogen interactions, despite the limited number of studies in this research area. Nevertheless, this review organizes existing knowledge and provides valuable insights into this underexplored aspect of *Leptospira* biology.

## 2. Diversity of Alternative σ Factors in Bacteria

**Figure 1:**
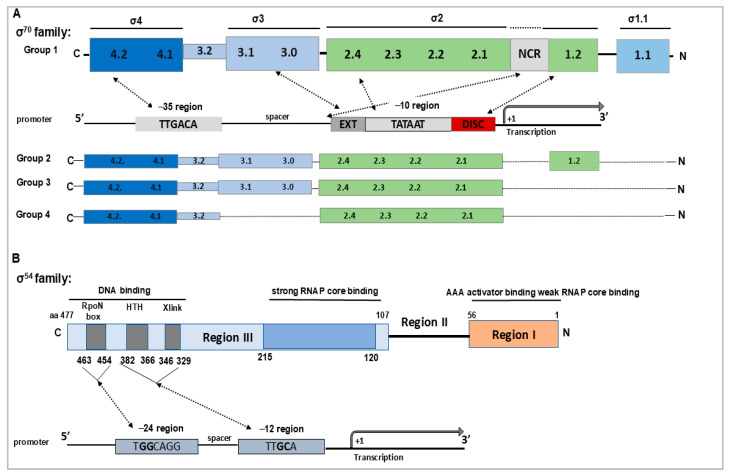
Schematic representation of the functional and structural domains of the σ^70^ and σ^54^ family of σ factors. (**A**) Domain organization of the four groups of σ factors within the σ^70^ family based on [18,23]. Key elements of the RNAP-σ^70^ recognized promoter are also indicated: −35 element, extended −10 element (T_−15_G_−14_N), −10 element (T_−12_ATAAT_−7_), and the discriminator sequence with the optimal trinucleotide motif 5′-GGG-3′, which is involved in regulation by the stringent response factor ppGpp during nutrient stress [20,24]. Recognition of these promoter elements is mediated by the corresponding σ^70^ domains, as indicated by arrows based on [18,20]. The σ2 and σ4 domains are critical for binding the RNAP core enzyme and initiating transcription. Group 1 σ factors additionally contain a non-conserved region (NCR) between σ1.2 and σ2.1 implicated in an interaction with promoter DNA just upstream of the −10 element, which facilitates DNA opening at the transcription start site (+1) [25]. (**B**) Functional domains of σ^54^ and their roles in transcription initiation based on [26]. Key motifs involved in promoter recognition are also indicated: DNA crosslinking motif (Xlink), a predicted helix-turn-helix (HTH) motif, and the RpoN box motif, all located within Region III. Region II, located between Regions I and III, is not essential for σ^54^ function, but its deletion—partial or complete—can significantly impair promoter recognition, DNA binding, and formation of the RNAP-σ^54^ holoenzyme [27].

Alternative σ factors belong to two evolutionarily distinct families: the σ^70^ family, which includes the primary housekeeping σ^70^-like factors, and the σ^54^ family, also known as RpoN. Although both families are essential for transcription initiation, they differ in domain architecture (Figure 1), promoter recognition, regulatory mechanisms, and biological roles, reflecting their specialized functions in bacterial physiology and pathogenicity [19,20]. Members of the σ^70^ family typically contain several well-conserved regions (σ1.1–σ4), which mediate core RNA polymerase binding, promoter recognition, and DNA melting (Figure 1A). In contrast, σ^54^ factors have a distinct domain architecture and require interaction with bacterial enhancer-binding proteins (bEBPs) to promote DNA melting and initiate transcription (Figure 1B) [18,20]. Most bacteria possess only one representative of the σ^54^ family, whereas the σ^70^ family includes multiple diverse members, reflecting their specialized roles in adaptation to environmental changes.

Promoters recognized by σ^70^-family proteins bound with core RNAP, i.e., the RNAP-σ^70^ holoenzyme, typically contain −35 and −10 elements, and transcription initiation proceeds directly upon RNAP binding, without additional activators. In contrast, the σ^54^ family, recognizes distinct −24/−12 promoter elements and strictly requires bEBPs, which use ATP hydrolysis to drive DNA melting and initiate transcription (Figure 1) [18,20,21]. This energy-dependent activation enables σ^54^ to regulate highly specialized and often energy-intensive pathways, such as nitrogen assimilation, motility, or host adaptation in bacterial pathogens. Functionally, σ^70^ family members provide broad transcriptional flexibility, from basal gene expression to stress adaptation, supporting rapid responses to environmental changes.

Within the σ^70^ family, four major groups have been defined based on structural and functional characteristics [16,22] (Figure 1A): group 1 includes the housekeeping σ^70^-like factors responsible for basal transcription; group 2 (e.g., σ^S^/RpoS) encompasses related but truncated forms governing stress adaptation and stationary phase adaptation; group 3 includes specialized regulators such as σ^28^ (FliA), controlling flagellar synthesis and motility, and σ^32^ (RpoH), which mediates the heat shock response; and group 4, which is the most diverse and numerous, is comprised of extracytoplasmic function ECF σs. Group 4 factors consist primarily of σ2 and σ4 domains, making them the smallest and most specialized σ factors. They regulate extracytoplasmic stress responses and are often controlled by cognate anti-σ factors, typically membrane-bound proteins that transmit extracellular or periplasmic signals to the cytoplasm.

Overall, the σ^70^ and σ^54^ families illustrate how bacteria balance broad transcriptional flexibility with pathway-specific regulation, which is crucial for adaptation and virulence in bacterial pathogens, including *L. interrogans.*

## 3. Alternative σ Factors in *L. interrogans*

### 3.1. σ^54^ (RpoN): Metabolic Adaptation and Virulence Potential

Genome-wide in silico analyses conducted by Zhukova et al. [15] identified canonical σ^54^-binding motifs in the promoter regions of two predicted lipoprotein genes (*LIC_12503* and *LIC_11935*) and the ammonium transporter gene *amtB* (*LIC_10441*), which is essential for growth under low-ammonium conditions. These motifs closely resemble the *E. coli* conserved σ^54^ promoter elements at positions −24 and −12, as validated by EMSA experiments [15,28]. Additional analyses using Proscan software revealed putative σ^54^-type promoters upstream of genes involved in glutamine biosynthesis (*glnA*), alanine racemization (*alr*), flagellar assembly (*fliO*), sodium/bile acid transport (*ncP1*), and lipid A biosynthesis (*lpxC*), suggesting that σ^54^ may have broader roles in *Leptospira* [28].

**Figure 2:**
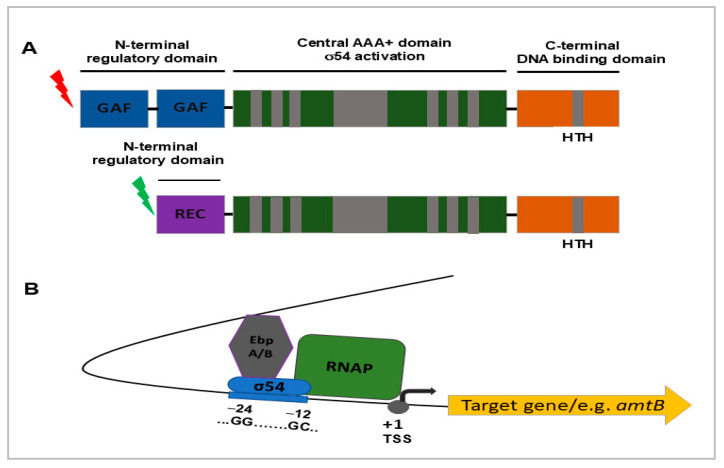
Schematic representation of domain organization of the EbpA/B transcriptional activators and the predicted EbpA/B-σ^54^ regulatory mechanism in pathogenic *L. interrogans* based on [28,29]. (**A**) Both EbpA/B activators (AAA+-type ATPases) of σ^54^-dependent transcription are composed of three functional domains: an N-terminal domain that is a “signal receiver”, a central AAA+ domain responsible for σ^54^ activation, and a C-terminal DNA-binding domain with an HTH (helix-turn-helix) motif that directs EbpA/B to a specific activation region in DNA. EbpA and EbpB have different regulatory/sensory domains and respond to different signals. EbpA contains two putative GAF (cGMP-specific phosphodiesterases, Adenylyl cyclases and FhlA) domains at its N-terminus, while EbpB contains a REC (receiver) domain at its N-terminus that is activated by phosphorylation by a cognate histidine kinase. The central domains of EbpA/B contain seven conserved regions essential for σ^54^-dependent transcription. (**B**) Different signals received and transmitted by the N-terminal sensory domain of EbpA/EbpB to the central AAA+ domain trigger their ATPase activity, oligomerization and transcription activation of a specific set of σ^54^-dependent genes. RNAP—RNA polymerase core enzyme. +1 TSS—transcriptional start site.

It is known that transcription initiating from σ^54^-dependent promoters requires ATP-hydrolyzing EBPs, which remodel the promoter region to facilitate transcription initiation [20]. The *L. interrogans* genome encodes two such regulators, EbpA (FhlA-like; LIC_10132) and EbpB (NtrC-like; LIC_11549), whereas saprophytic *Leptospira* species encode only EbpA [14,28]. It has been proposed that EbpA is mainly associated with transcription of genes whose products support environmental survival in both pathogenic and saprophytic species, while EbpB may provide functions relevant to host adaptation in pathogens [14]. Both activators exhibit modular organization, comprising an N-terminal sensory domain, a central AAA+ ATPase domain required for σ^54^ activation, and a C-terminal helix–turn–helix (HTH) DNA-binding domain that targets enhancer elements (Figure 2) [28,29]. This dual regulatory system enables *L. interrogans* to respond to diverse environmental signals and coordinate σ^54^-dependent transcription.

Recent experimental evidence indicates that the EbpA–σ^54^ pathway in *L. interrogans* regulates expression of at least three genes, including *amtB* (an ammonium transport protein gene) and two putative lipoprotein-encoding genes [28]. It was demonstrated that recombinant RpoN and EbpA specifically bind to the promoter and upstream regions of these genes, respectively. Furthermore, genetic disruption of *ebpA* and subsequent complementation experiments confirmed that this pathway is required for transcription of the three mentioned genes, resulting in impaired growth under nitrogen-limited conditions and markedly reduced survival in water, whereas virulence in animal infection models remained unaffected. Thus, although this regulatory system is not essential for infection of mammalian hosts, it plays a crucial role in bacterial survival in aquatic environments, emphasizing its importance for environmental adaptation during the leptospiral enzootic cycle [28]. Notably, in the saprophytic species *L. biflexa*, σ^54^ directly controls a newly identified nitrogen-responsive gene, LEPBI_I1011, and is necessary for long-term environmental persistence [29].

Taken together, in *L. interrogans*, the EbpA–σ^54^ pathway primarily supports environmental survival and nitrogen metabolism, complementing observations in other spirochaetes (particularly in *Borrelia burgdorferi*) where similar systems contribute to host adaptation, virulence, and mammalian host infection [30,31,32]. It is worth noting that in *B. burgdorferi*, the response regulator Rrp2 is the sole EBP that activates RpoN (σ^54^)**,** thereby initiating the RpoN–RpoS regulatory cascade essential for mammalian infection. This pathway governs expression of key virulence factors, such as OspC, DbpA, and Mlp8, and is crucial for *B. burgdorferi* survival and adaptation during its enzootic cycle [30,33]. Moreover, acetyl phosphate is suggested to activate the Rrp2-RpoN-RpoS pathway, supporting the concept that this molecule can serve as a global signal in bacterial pathogenesis [34].

### 3.2. σ^28^ (FliA): Motility Control and Contribution to Virulence

Genomic analyses have revealed that *L. interrogans* encodes a σ^28^ factor (FliA) [35]. It is likely involved in the regulation of late flagellar and chemotaxis genes, consistent with its role in other spirochaetes, with the exception of *Borrelia*, which lacks σ^28^. Supporting this hypothesis, genome-wide mapping of transcription start sites in *L. interrogans* identified σ^28^-binding motifs upstream of genes encoding structural components of the leptospiral endoflagellum, including sheath protein FlaA1, core proteins FlaB1 and FlaB4, and a flagellin-specific chaperone FliS [15]. The endoflagellum is indispensable for motility, enabling *Leptospira* to penetrate blood and tissues, thereby facilitating tissue penetration and dissemination within the host [36,37,38]. Notably, FlaA1 is required not only for motility but also plays a key role as a virulence factor during leptospirosis pathogenesis. Its loss disrupts flagellar assembly, leading to non-motile mutants with markedly reduced infectivity in animal models [37]. In addition, up-regulation of *fliS* expression (~1.98-fold) in *L. interrogans* cells upon exposure to normal guinea pig serum suggests a protective role of its product (FliS) against stress-induced aggregation and degradation of flagellar proteins [39]. Collectively, these findings indicate that σ^28^ may contribute to gene regulation during the early phase of spirochetemia infection.

More recently, it has been shown that in another pathogenic spirochaete, *Treponema denticola,* activity of σ^28^ is regulated by FlgM, an anti-σ factor that inhibits transcription of the late flagellar genes [40]. This discovery has expanded our understanding of σ^28^ regulation in spirochaetes. Remarkably, the *T. denticola* FlgM regulator displays distinct functional properties compared to its homologs in other bacteria: (i) it stabilizes FliA by preventing its degradation; (ii) *flgM* deletion results in reduced, rather than elevated, flagellin gene expression; (iii) it interacts with other regulators such as CsrA; and (iv) its lack alters flagellar number and polarity, leading to impaired motility. Sequence-based searches using the *T. denticola* FliA and FlgM as queries revealed FlgM-like genes in *Leptospira* spp., indicating the presence of a potential anti-σ factor homolog [40]. However, no direct evidence currently supports the presence of a FlgM homolog in *L. interrogans*. Thus, the existence of an anti-σ factor controlling σ^28^ activity in *L. interrogans* remains to be experimentally investigated.

Taken together, these observations suggest that σ^28^ in *L. interrogans* may play a similar role in controlling genes essential for motility and virulence, reflecting a conserved regulatory strategy among spirochaetes that links flagellar assembly to environmental sensing and pathogenesis.

### 3.3. ECF σ Factors: Diversity, Regulatory Mechanisms and Predicted Functions

Genome-wide dRNA-seq analyses have predicted the presence of 469 putative σ^24^-binding sites in the promoter regions of *L. interrogans* genes, which may be recognized by ECF σ^E^-type factors [15]. It should be noted that σ^24^-dependent promoters often exhibit poorly conserved −35 elements in phylogenetically distant bacteria, making accurate prediction of σ^24^ targets in *L. interrogans* challenging. Interestingly, the number of ECF σs varies among *Leptospira* species: pathogenic *L. interrogans* encodes 11 ECF σs, whereas saprophytic species contain only five [14]. Most of these ECF σ-coding genes are located on the large chromosome (CI), with one on the small chromosome (CII) (Table 2). Based on ECF σ classification performed using the ECF hub web page (an interactive data platform for ECF σ factors) and following Casas-Pastor et al. [41], *L. interrogans* ECF σ factors were clustered into five groups: ECF208 (2 factors), ECF242 (2 factors), ECF16 (2 factors), ECF229, and ECF246 (one factor each), with three additional ECF σs remaining unclassified (Table 2) [18]. Two ECF σs, i.e., LIC_12490 and LIC_10144, remained ungrouped in the new ECF classification, while another ECF σ factor, LIC_10225, was not included in this re-classification and remained unclassified under the new criteria [18].

**Table 2:** ECF σ factors (σ^E^-type) from *L. interrogans* based on [18].

^a^ Gene ID	ECF Group	ECF Subgroup	NCBI Reference Sequence (Protein ID)	Number of Amino Acid Residues	Calculated Molecular Weight
	Chromosome I (large)				
*LIC_10144*	ungrouped	unsubgrouped	WP_000777857.1	174	20,096
*LIC_10225*	unclassified	unclassified	WP_001054050.1	301 (21-194 σ^70^-ECF family region)	35,314
*LIC_10386*	ECF242	ECF242s1	WP_000951509.1	182	20,970
***LIC_10559***	ECF208	ECF208s1	WP_000988152.1	181	21,087
*LIC_10644*	ECF229	ECF229s2	WP_000777857.1	174	20,321
*LIC_11817*	ECF16 (SigF-like)	ECF16s14	WP_001274737.1	184	21,620
^b^ *LIC_12490*	ungrouped	ECFs21	WP_000378482.1	206	23,881
***LIC_12757***	ECF229	ECF229s1	WP_000081452.1	180	21,105
*LIC_13266*	ECF246	ECF246s2	WP_001209037.1	192	22,906
*LIC_13285*	ECF208	ECF208s2	WP_000435437.1	169	19,550
	**Chromosome II (small)**				
*LIC_20115*	ECF242	ECF242s2	WP_001971886.1	166	19,377

Note: ^a^ Gene IDs are based on ORFs from the genome sequence of *L. interrogans* serovar Copenhageni deposited in GenBank under accession numbers AE016823 (chromosome I; 4,277,185 bp) and AE016824 (chromosome II; 350,181 bp) [35]. Genes in bold were found to be up-regulated at elevated temperatures [42]. ^b^ ECF present in both the pathogenic *L. interrogans* and the saprophytic *L. biflexa*.

Specific functions of individual ECF σs in both pathogenic and saprophytic species remain largely unknown. It has been proposed that ECF σs unique to pathogenic *Leptospira* contribute to survival and adaptation within mammalian hosts [14]. The above-mentioned re-classification has provided new insights into the potential regulatory mechanisms and functional roles of *L. interrogans.* ECF σs. Potential functions of these factors include proton-motive force-dependent ion transport (ECF229), detoxification of harmful compounds (ECF242), antibiotic resistance (ECF246), and responses to heavy metal or oxidative stress (ECF16) (Table 2). Notably, two ECF of these σ factors, i.e., LIC_12757 (ECF229s1) and LIC_10559 (ECF208s1), were found to be 1.5-and 2-fold up-regulated at higher temperatures, respectively [42]. LIC_10559 is particularly noteworthy due to its exclusive presence in highly pathogenic *Leptospira* spp. [14]. We have recently demonstrated that LIC_10559 is able to activate the host’s immune system and elicit a specific antibody response in infected animals [43], providing direct evidence of its functional role during infection. These findings strongly support the involvement of LIC_10559 in leptospiral pathogenesis and highlight its potential as a promising candidate for future diagnostic applications. Furthermore, we have shown that LIC_12757 may regulate, especially under thermal stress, transcription of *L. interrogans clpB* gene encoding the AAA+ chaperone ClpB [44]. Of note, this chaperone, acting as a disaggregase in bacterial cells [45], is a leptospiral virulence factor [46] and belongs to *L. interrogans* hub proteins interacting with human proteins [47]. It is important to note that interactions between bacterial proteins and the host are critical for successful evasion of the host’s immune defenses. Consequently, it is plausible that LIC_12757 regulates transcription of genes that enhance bacterial survival under stress and facilitate escape from the host immune responses [18,43]. On the other hand, overexpression of LIC_10144 (ungrouped ECF σ) has similarly elevated *clpB* promoter activity, indicating that multiple ECF σs may coordinate stress responses in *L. interrogans* [44].

**Figure 3:**
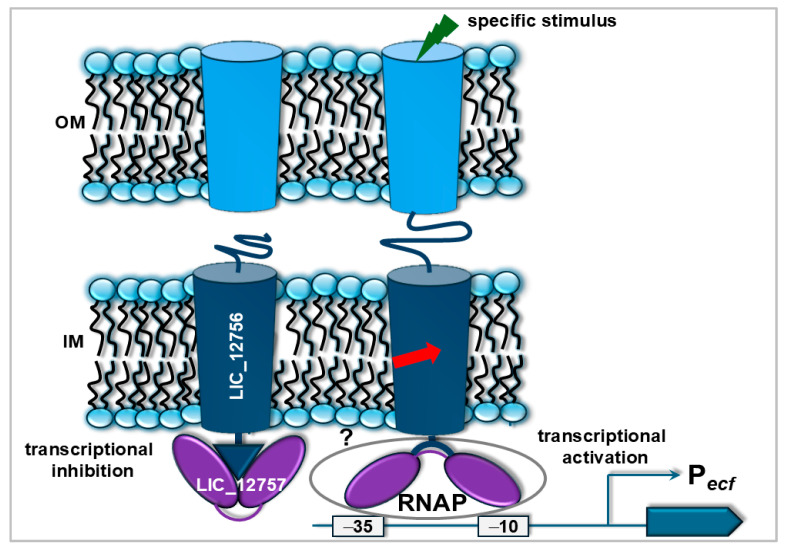
The proposed model of LIC_12757 regulation mediated by interactions with LIC_12756 is based on [49]. This schematic illustrates the dual regulatory effect of LIC_12756 on the transcriptional activity of LIC_12757 under different environmental conditions. In the absence of a specific external signal, LIC_12756 inhibits the activity of LIC_12757, preventing it from carrying out its σ factor function (transcriptional inhibition). Conversely, when an appropriate environmental stimulus is present (e.g., iron limitation), LIC_12756 activates LIC_12757, possibly by promoting its interaction with the RNA polymerase core enzyme (RNAP). This interaction facilitates RNAP recruitment to target promoters, initiating gene transcription (transcriptional activation). The arrow indicates a potential processing event of the leptospiral FecR-like regulator at the membrane, involving intramembrane proteolysis and release of its N-terminal cytoplasmic fragment together with the associated σ factor [50]. The light blue cylinders denote unidentified receptors involved in both transport and signal transduction (e.g., iron uptake receptors). OM—outer membrane; IM—inner membrane.

Our recent studies have also provided insights into regulatory mechanisms of ECF σs in *L. interrogans* [48,49]. First, we have demonstrated that LIC_12757 is autoregulated at the transcriptional level [48]. Further, our investigations revealed that LIC_12757 interacts with a putative FecR-like regulator, LIC_12756, which functions as both an anti-σ factor and a positive regulator of LIC_12757 under specific environmental conditions [49]. Interestingly, our results suggest that nutrient-limiting conditions, including iron deficiency, may act as specific signals for the LIC_12757activation. As these conditions are also encountered by *Leptospira* during host infection, it is likely that the LIC_12757–LIC_12756 regulatory system is active under these conditions, controlling expression of a subset of genes essential for bacterial survival within the mammalian host. Precise identification of these target genes will require further studies. According to our recently proposed regulatory model [49], under inducing conditions, LIC_12756 promotes LIC_12757 binding to the RNA polymerase core enzyme to initiate transcription and may undergo membrane-associated processing, releasing its N-terminal fragment together with the associated σ factor LIC_12757 (Figure 3) [50]. We have also shown that LIC_12756 may not only assist LIC_12757 in binding to the RNA polymerase core enzyme, but also stabilize LIC_12757 by preventing its proteolytic degradation. It is also worth mentioning here that its similarity to the *E. coli* FecI–FecR system suggests that the leptospiral LIC_12757–LIC_12756 regulatory system could involve a TonB-dependent receptor, such as a FecA-like protein, potentially transmitting extracellular signals to LIC_12756 and subsequently to LIC_12757. Nevertheless, previous comparative and functional genomic studies have indicated that none of the predicted TonB-dependent receptors in *Leptospira* contain the N-terminal extension typical for interaction with an anti-sigma factor, as observed in the *E. coli* FecI–FecR system [51]. Despite this, exploring such a possibility seems worthwhile.

Overall, a regulatory system in *L. interrogans*, consisting of the LIC_12757 ECF σ factor and the LIC_12756 FecR-like regulator, represents one of this bacterium’s strategies to appropriately adjust gene expression in response to host-induced stress.

It is worth noting that the *L. interrogans* genome encodes over 30 predicted regulatory proteins, including anti-σ and anti-anti-σ factors, and their modulators, which may influence ECF σ factor activity in response to environmental stimuli [14] as listed in the review by Kędzierska [18]. Additional regulatory inputs may come from two-component systems (2CS), of which *L. interrogans* possesses 74 genes, reflecting a complex network of environmental sensing [14].

Further studies are needed to elucidate the specific roles of each predicted ECF σ factor. In particular, comprehensive functional analyses will be essential to clarify how these factors contribute to leptospiral adaptation under environmental stresses and during mammalian host infection. Elucidating their diverse activities will not only enhance our understanding of leptospiral physiology but may also help identify potential targets for diagnostic or therapeutic applications.

## 4. Concluding Remarks and Future Perspectives

The presence of σ^54^, σ^28^, and multiple ECF σs highlights the layered complexity of transcriptional control in the pathogenic *L. interrogans*. These alternative σ factors likely act at different stages of the leptospiral life cycle: σ^28^ is engaged in motility and host entry, σ^54^ in metabolic adaptation, and ECF σs in environmental stress survival and possibly virulence. The regulatory pair LIC_12757–LIC_12756 demonstrates how a specific ECF σ factor and its cognate regulator may mediate responses to iron and nutrient limitation, as well as to host-induced stress, allowing the pathogen to adjust gene expression accordingly. Unraveling the interplay between these regulatory networks remains a major challenge, but its understanding could reveal novel strategies used by *Leptospira* to thrive in diverse environments and cause disease.

Despite advances in comparative genomics and bioinformatic predictions, functional understanding and characterization of alternative σ factors and regulatory elements in *L. interrogans* remains incomplete. The development of more efficient genetic tools, including CRISPR-based mutagenesis and transcriptional reporters, as well as RNA-seq analyses, will be essential to study genes regulated by σ^54^, σ^28^, and ECF σs. Characterization of specific ECF group regulons, such as ECF242 (FecI-like), ECF229, and ECF208, may help to uncover novel pathways involved in stress adaptation, environmental persistence, and host colonization. The use of the guinea pig model and the murine macrophage-like cell line J774.1 as an in vitro model could facilitate the investigation of the role of ECF σs in *Leptospira* virulence and host–pathogen interactions. It has been demonstrated that this cell line is a permissive host cell for virulent leptospires [52]. Moreover, structural and biochemical studies on interactions between ECF σs and their anti-σ partners could provide new insights into the unique regulatory mechanisms of spirochaetes. A deeper understanding of these regulatory elements in *L. interrogans* will not only advance our knowledge of bacterial transcriptional control but also lay the groundwork for novel strategies to combat leptospirosis.

## Figures and Tables

**Table 1:** Summary of σ factors present in pathogenic *L. interrogans* and their corresponding σ factors in *E. coli* based on [18].

σ Factor/Group/Gene Locus	Predicted Number	Proposed Function/Controlled Genes	Evidence/Characterization	Representative Homolog (*E. coli*)
σ^70^ family				
σ^70^/group 1 *LIC_11701*	1	controls basal transcription; essential for growth and basic cellular functions/mainly housekeeping genes (>1000 genes)	supported by genomic and expression data	RpoD (σ^70^)
σ^28^/group 3 *LIC_11380*	1	directs motility and flagellar gene regulation; contributes to virulence/genes encoding components of the endoflagellum (*flaA1*, *flaB1*, *flaB4*) and the flagellin-specific chaperone FliS (*fliS*)	predicted by comparative genomics	FliA (σ^28^)
ECF (σᴱ-type)/ group 4 (see Section 3.3)	11	mediates responses to extracytoplasmic and envelope stresses, contributing to adaptation and virulence/469 putative binding sites in the *L. interrogans* genome	predicted by genomic analyses; biochemically uncharacterized, except for LIC_12757 and LIC_10559	RpoE (σ^24^/σᴱ)
σ^54^ family				
σ^54^ *LIC_11545*	1	regulates nitrogen metabolism and complex cellular processes, contributing to environmental adaptation/genes encoding putative lipoproteins and the ammonium transporter AmtB; might be involved in glutamine synthesis (*glnA*), alanine racemization (*alr*), flagellar synthesis (*fliO*), sodium bile acid symport (*ncP1*), and lipid A biosynthesis (*lpxC*)	predicted by comparative genomics; studied *in vitro*	RpoN (σ^54^/σ^N^)

## Data Availability

No new data were created or analyzed in this study. Data sharing is not applicable to this article.

## Abbreviations

The following abbreviations are used in this manuscript:

EBP	enhancer-binding protein
ECF σs	extracytoplasmic function σ factors
*L. interrogans*	*Leptospira interrogans*
RNAP	RNA polymerase core enzyme
